# An educational intervention using the health belief model for improvement of oral health behavior in grade-schoolers: a randomized controlled trial

**DOI:** 10.1186/s12903-022-02132-2

**Published:** 2022-03-27

**Authors:** Hormoz Sanaeinasab, Mohsen Saffari, Hassan Taghavi, Aliakbar Karimi Zarchi, Fatemeh Rahmati, Faten Al Zaben, Harold G. Koenig

**Affiliations:** 1 Health Research Center, Life Style Institute, Baqiyatallah University of Medical Sciences, Tehran, Iran; 2 Health Education Department, Faculty of Health, Baqiyatallah University of Medical Sciences, Tehran, Iran; 3 Student Research Committee, Baqiyatallah University of Medical Sciences, Tehran, Iran; 4 Department of Epidemiology and Biostatistics, Baqiyatallah University of Medical Sciences, Tehran, Iran; 5 Department of Medicine, King Abdulaziz University, Jeddah, Saudi Arabia; 6 Department of Psychiatry and Behavioral Sciences, Duke University Medical Center, Durham, NC USA; 7 School of Public Health, Ningxia Medical University, Yinchuan, China

**Keywords:** Oral health, Dental caries, Health education, Behavior, Gingival health

## Abstract

**Background:**

Oral health problems especially dental caries are common in school children, and education programs may help to prevent these conditions. The aim of current study was to examine the effects of an educational program based on a Health Belief Model (HBM) to improve oral health behaviors of elementary school children.

**Methods:**

A total of 112 children ages 6–12 years old accompanied by one of their parents were randomly assigned to intervention/test and control groups. In the intervention group, five consecutive weekly educational sessions based on the HBM were provided, while the control group received only routine education delivered by the dental clinic. The Decayed, Missing, and Filled Teeth (DMFT) score, papillary bleeding index, and responses to the HBM questionnaire were assessed in the intervention and control groups at baseline and three-month follow-up after the intervention was completed. Within-group and between-group differences were examined using the Student’s t-test and analysis of covariance.

**Results:**

All HBM domains were improved at follow-up in the intervention group compared to the control group (p < 0.001). The largest change was in perceived susceptibility, whereas the smallest changes were in perceived severity and perceived benefits. The papillary bleeding index demonstrated a significant change from baseline to follow-up in the intervention group (reduction of 0.7, 95% CI = − 0.9 to − 0.5). All components of the DMFT score except missing teeth also improved in the intervention group compared to controls. However, no significant difference was found in total DMFT score between intervention and control groups.

**Conclusion:**

An education program based on HBM may be more effective than current methods used to educate children and their parents on optimal oral health behaviors. Administration of interventions of this type along with other school-based programs to prevent dental caries may be helpful in grade-school children.

## Background

Oral health is an integral part of physical and mental health and has been found to be a strong determinant of quality of life and well-being in general populations [1, 2]. According to World Health Organization (WHO), oral health is a state where individuals are free from pain in the facial area and oral cavity and are without problems such as malignancy, infection, periodontal disease, or tooth decay/loss which limit the normal functioning of chewing, smiling and talking [3]. Dental caries have been recognized as a common condition among school-age children [4]. Over one-third of world’s population are affected by oral health problems, and children are particularly vulnerable, especially with regard to untreated caries [5, 6]. In the Unites States, more than half of grade-schoolers (children attending elementary school) suffer from tooth decay, and the situation in middle and low income countries is even worse, with more than 90% of children in this age group experiencing dental caries [7–9]. The prevalence of oral diseases such as gingivitis in school-age children of countries such as Turkey and Iran has been estimated to be between 70 and 75% [10, 11], and poor oral hygiene has been found in 45% of children attending primary schools in Bangladesh [12].

Children with poor oral health are nearly 12 times more likely than healthy children to suffer from restrictions in daily activities [13]. Not only do these children experience problems related to oral diseases such as gum bleeding, inflammation and toothache, but also suffer from sleeping problems, school absence, decreased academic achievement, and changes in dietary habits (with consequent weight loss and developmental delay) [14–16]. Children with oral health problems also experience speech problems, irritability, low self-esteem, mental health problems, difficulties in learning, a variety of inflammatory diseases, and increased hospitalization [17–19]. Most of these problems are preventable because they are lifestyle-dependent conditions, and modifying oral health behaviors during childhood plays a critical role in improving oral health as children grow older [20].

Oral health promotion programs are reported to be the best strategy for overcoming oral health problems in school-age children [21]. However, most interventions of this type are limited to school-based programs. Using the school as an appropriate setting for oral health promotion has been suggested by WHO, and health education on oral issues, supervised tooth brushing, fluoride therapy, and application of fissure sealant have been found to be useful for school-age children in the school setting [22]. However, educational interventions in health care centers and oral health clinics should also be considered. Although the effectiveness of educational interventions in such settings to promote oral health has been examined in several studies [23], there is still a research gap regarding theory-based health education programs in the clinical setting.

Childhood, especially during the grade-school years, is one of the most important times to deliver oral health education because interventions at this time may lead to the development of habits that continue into adulthood, resulting in a lifetime of oral health benefits [24]. Research indicates that children as young as 6–12 years old have the ability to assimilate information leading to beliefs that influence their conscious choices with regard to health [25, 26]. However, most oral health programs today are concentrated on adolescents. Since oral health habits mostly develop during childhood, employing oral health education interventions at this developmental stage may lead to better outcomes than providing such education at older ages. Increasing child (and parent) awareness of their susceptibility to dental caries and instilling positive attitudes toward oral health behaviors are key elements in promoting a healthy lifestyle, which may be accomplished through interventions based on health education theories/models [27]. For example, a systematic review on strategies to prevent dental caries in children has underscored the importance of utilizing theory based educations to improve oral health status during childhood [28].

The Health Belief Model (HBM) is a common and widely used health education approach to explain and predict preventive behaviors. According to the HBM as applied to oral health education, when individuals find themselves susceptible to oral health problems and understand the severity of such problems, they will be more likely to adopt recommended oral health behaviors. Other aspects of the model, such as perceived benefits vs. perceived barriers, responding to cues to action expressed in the media, and individual confidence in their ability to taking action in disease prevention (self-efficacy) contribute to the changing of unhealthy behaviors and adoption of healthy ones [29]. Previous studies of the HBM for improving oral health have shown that such interventions not only positively influence self-reported oral health practices, but also improve clinical parameters such as DMFT and gingival bleeding index scores [30–32]. Nevertheless, evidence on the effectiveness of such programs in school-age children is limited. Therefore, this study sought to assess the effectiveness of a health education program based on HBM to promote oral health behavior in elementary school children.

## Methods

### Sample and procedures

A randomized controlled trial was conducted in elementary school children and their parents seen between May and August 2017 in a dental clinic located in a middle class socioeconomic area of Tehran, Iran. Children were recruited from a public dental clinic that provides dental care services mostly to those covered by health insurance. The majority of services in this clinic are free or at low cost to patients. A convenience sample fulfilling inclusion and exclusion criteria was recruited to participate in the study. Sample size was determined using a formula suggested by Chow et al. [33] to estimate the number of participants necessary to identify at least a moderate effect size of 0.55 for behavior change [34] with a type I error rate of 0.05 and type II error rate of 0.20 for a one unit change in standard deviation. With an estimated attrition rate of 5 percent, it was determined that 112 participants would be necessary to include in the study. Therefore, this number of participants was identified and randomly assigned to either the intervention or control group using a random numbers table with an allocation ratio of 1:1. Simple randomization was performed using a shuffled deck of cards. Sequentially numbered cards were used to perform determine the random allocation sequence. Enrolling participants and random assignment to intervention or control groups was carried out anonymously by individuals on the research team. Because of the nature of intervention (education), there was no possibility of blinding examiners, who knew which group participants were in. Only statistical analyses were performed blinded to treatment group. Inclusion criteria were age 6–12 years (elementary school children), participation of at least one parent (either father or mother), being a permanent resident in the enrollment area, having a medical record in the dental clinic, and being able to speak and understand Persian. Children with advanced oral diseases or other serious medical conditions or disabilities, those routinely using anti-inflammatory agents, those who had used antibiotics within the past two weeks, children with illiterate parents or parents whose job was related to dentistry, and those receiving ongoing orthodontic treatments were excluded. The main outcome was oral health status as measured by DMFT score, gingival health index, and constructs of the HBM and related behaviors. The dentist who performed the clinical examination was first educated on the study’s aims and the nature of intervention. She then, accompanied by an oral health educator, conducted the examination and answered any queries regarding the scale. The training was done based on WHO guidelines [35, 36] however, no calibration was performed to calculate Kappa correlation index for caries examination.

**Fig. 1:**
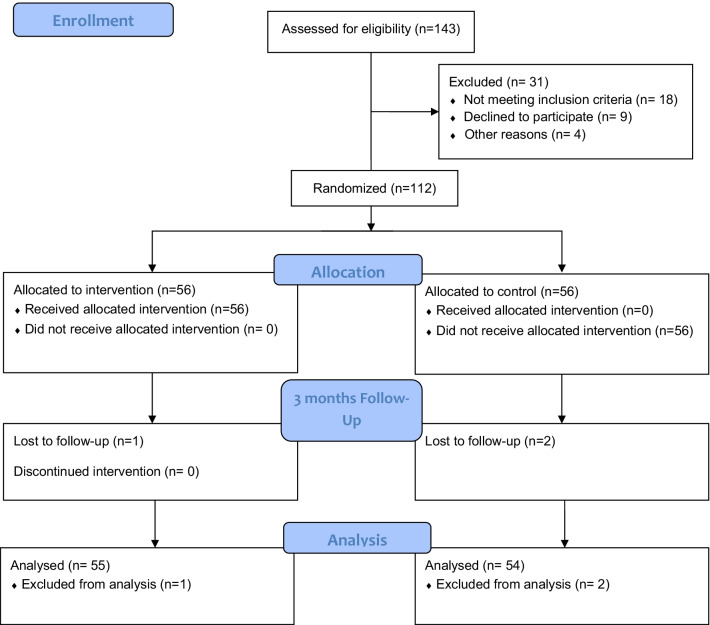
CONSORT flow diagram of the study

The study protocol was approved by the institutional review board committee of Baqiyatallah University of Medical Sciences (#BMSU.1395.63789). The study was also retrospectively registered on 08/11/2021 in the clinicaltrials.gov under this identifier: NCT05112224. Children and their parents were informed about the study and provided written informed consent to participate (written consent from parents and oral consent from children). Administrative staff in the dental clinic, after being informed on study qualifications, directed potential participants to researchers, who provided further information about the study and obtained consent. Those who met inclusion criteria then completed questionnaires and underwent a dental examination to be described later. The dental examination was conducted by a trained dentist. Questionnaires were distributed and explained by a qualified health educator. Parents completed the demographic and HBM-related questionnaires. The CONSORT flow diagram of the study has been included in the Fig. 1.

### Measures

Demographic information on child and parent age, academic grade, parent education, parent job, family income level, and coverage for dental services by health insurance was obtained from parents.

### Papillary bleeding index (PBI)

The PBI was used for gingival assessment. This scale, developed by Muhlemann (1977), was used to evaluate different areas of gingiva in terms of bleeding when probed [37]. This was conducted by entering a probe into the base of papilla and then moving it to the tip of the papilla. The distal aspect of the papilla was also assessed in this way. PBI ratings consist of 5 grades. Grade 0 indicates no bleeding on probing, whereas grade 4 indicates profuse bleeding that spreads to the marginal gingiva. The scale has been used in previous studies in Iranian populations and demonstrated acceptable psychometric properties in diagnosing gingivitis and periodontal problems.

### Decayed, missing, and filled teeth (DMFT)

DMFT is a traditional oral health index developed in 1930s and is still used as the primary measure of dental health around the world, especially for epidemiological purposes. The index provides the sum of decayed, missing, and filled teeth that an individual has, and ranges from 0 to 28 in adults, with higher scores indicating poorer oral health status. When a person is caries free, the DMFT score is 0. Number of decayed, missing and filled teeth may also be calculated separately, with the proportion of decayed teeth (D/DMFT) indicating unmet treatment need and the proportion of filled teeth (F/DMFT) indicating access to dental services. DMFT score is also one of the common oral health indices used in young Iranians, especially among school-age children. The DMFT score in 12-yer-old children is one of the standard measures of oral health used by WHO to compare oral health between regions and countries [36]. To measure DMFT score the study examiner (dentist) must be trained using guidelines provided by the WHO that have been manualized [36]. The DMFT scale has been used in several experimental studies to measure changes related to oral health [21].

### Health belief model questionnaire (HBMQ)

The HBMQ developed by Kasmaei et al. (2014) addresses six aspects of HBM, including subscales assessing perceived susceptibility (3 items), perceived severity (7 items), perceived benefits (3 items), perceived barriers (7 items), self-efficacy (5 items), cues to action (3 items), and behavior (3 items). Responses to items on all subscales, except the behavior subscale, range from completely disagree (0) to completely agree (4), asking participants to rate sentences representing their beliefs concerning each of these six domains. The behavior subscale asks about frequency of behaviors such as use of mouth wash, teeth brushing, and teeth flossing from never (0) to always (4). The score of each subscale is computed by summing the score of all items belonged to that subscale and for the total score, summing scores on all subscales with the exception of the perceived barriers subscale. Higher subscale and total scores indicate more positive attitudes/beliefs. For the perceived barriers subscale, lower scores indicate that the person believes they have the capacity to overcome barriers related to improvement of his/her oral health status. The HBMQ was originally developed as a part of a doctoral thesis in Iran to measure HBM constructs on oral health in Iranian elementary school children and their parents, and has well-established psychometric properties in this population [38].

### Educational intervention

Those in the test group received an educational intervention as described below, while those in the control group received only the routine educational program provided by the dental clinic. The routine educational program for control group consisted of a weekly face-to-face sessions on the importance of oral health for children and related preventive behaviors such as brushing and flossing. This education was provided by an oral health technician over a time period of approximately one hour, which is largely repetitive from session to session. The educational program for the test group was scheduled in five consecutive weekly sessions based on HBM principles. HBM principles including perceived susceptibility, perceived severity, perceived benefits and barriers, cues to action, and self-efficacy were used to design the intervention program. The intervention was applied by an oral health educator (expert both on oral health and health education). The conceptual theoretical approach for this intervention was adapted from Glanz et al. (2015) [29]. Each session lasted approximately one hour, and all of sessions were held between 9:00 and 11:00AM. Participants in the intervention group (children and one of their parents) were divided into groups consisting of 12–15 members in whom the educational program was administered. As noted above, the trained health educator skilled in oral health education facilitated all of the sessions.

In the first session, focus was placed on perceived susceptibility. For this session, a short lecture (20 min) was first provided that described statistics on oral health diseases especially for dental caries in school-age children. Next, participants in the group were asked to explain how much they thought they might be at risks of such problems and why. Both children and parent were encouraged to participate in this discussion. In the second session, the focus was on perceived severity. This session began by describing a story on outcomes related to oral health problems. In this story, the experiences of a 10-year-old boy regarding health problems due to dental caries were described, thereby illustrating problems that may develop for children who failed to engage in preventive strategies and early treatment of dental problems. At the end of this session participants were encouraged to share first-hand or second-hand experiences that may have been similar to the case presented. The third session emphasized oral disease prevention skills and their perceived benefits. The behaviors most strongly emphasized were teeth brushing, flossing, and use of mouthwash, illustrated on a model of teeth. Information regarding the timing and frequency of such behaviors was also provided. Participants were then asked to prepare a list of all potential benefits regarding the demonstrated behaviors and then to discuss these benefits with the overall group. The fourth session focused on perceived barriers to performing oral health behaviors. Participants were asked to bring up and discuss attitudes or beliefs that might prevent them from carrying out the behaviors correctly. The facilitator, with contribution from other participants in the group, then corrected wrong beliefs and provided strategies for overcoming the barriers. In the fifth and last session, the health educator discussed how to increase self-efficacy with regard to oral health behaviors. First, an educational video was played showing a healthy role model, i.e., a child correctly performing behaviors such as brushing and flossing. Participants were then asked how much they trusted themselves to correctly perform these behaviors and if there were any questions on the proper ways to do so. Competing behaviors or stressors that might impede participants from performing the health-related behaviors were also asked about and suggestions on how to overcome those barriers were provided. Finally, to address the cues to action dimension of the HBM, the cell phone number of parents was obtained and a text message reminder was sent every two weeks for three months after the intervention on the importance of oral health behaviors.

### Data analysis

Descriptive data were reported by means (M) (with standard deviation) for quantitative measures or numbers (N) (with percent) for categorical variables. To examine between-group differences at baseline, the Chi-square test or Fisher’s exact test (if applicable) was used for categorical variables. Normal distributions were examined using the Shapiro–Wilk test. If the p value for this test is greater than 0.05, then the data distribution is considered normal. The Student’s t-test was also used to examine between-group differences before and after the intervention for continuous variables. The Levene’s test was used to assess the homogeneity of variances between groups when using the t-test. If the resulting p value on this test is greater than 0.05, then no significant difference between groups is considered present in terms of variances. The paired t-test was used to assess within-group differences from baseline to follow up. Means and 95% confidence intervals (CI) were used in reporting between-group and within-group differences. To adjust for baseline differences between groups, analysis of covariance test (ANCOVA) was used. In the model, baseline variables were entered as covariates with group as a fixed factor (independent variable) and follow-up data on dependent variables. Homoscedasticity was assessed before using ANCOVA by producing a scatter plot of residuals compared to predicted values. For significant results, the alpha level was set at less than 0.05. All statistical analyses were performed using SPSS version 24 for Windows (IBM statistics, Armonk, NY).

## Results

The mean age of children participating in the study was 9.8 years (SD, 3.4) and half (51%) were female. Greater than 60% of mothers in both groups were younger than 35 years, whereas approximately half of fathers were 35 years old or younger. The average education of fathers was greater than for mothers, and while the most of fathers were employed outside the home, the majority of mothers were home-keepers (> 0.80%). Nearly 80% of participants in both groups had health insurance with support for dental services. In terms of economic status, approximately 60% of parents reported their family income as moderate (neither good nor bad). There were no significant differences between groups at baseline on demographic characteristics (Table 1).

**Table 1:** Characteristics of intervention and control groups (n = 112)

Variables	Intervention group (n = 56)	Control group (n = 56)	Group comparison X^2^ (p value)
Age (years)			
6–9	23 (41.1)	19 (33.9)	0.609 (0.434)
9–12	33 (58.9)	37 (66.1)	
Sex			
Boy	26	29	0.321 (0.570)
Girl	30	27	
Mother’s age			
< 35	38 (67.9)	34 (60.7)	0.622 (0.430)
≥ 35	18 (32.1)	22 (39.3)	
Father’s age			
< 35	31 (55.4)	26 (46.4)	0.893 (0.344)
≥ 35	25 (44.6)	30 (53.6)	
Mother’s education			
University	22 (39.3)	27 (48.2)	0.907 (0.340)
High school or lower	34 (60.7)	29 (51.8)	
Father’s education			
University	35 (62.5)	42 (75.0)	2.036 (0.153)
High school or lower	21 (37.5)	14 (25.0)	
Mother’s job			
Employee	7 (12.5)	11 (19.6)	1.059 (0.303)
Housekeeper	49 (87.5)	45 (80.4)	
Father’s job			
Employee	47 (83.9)	53 (94.6)	3.36 (0.066) F value = 0.123^*^
Self-employed	9 (16.1)	3 (5.4)	
Health insurance with dental coverage			
Yes	43 (76.8)	49 (87.5)	2.191 (0.138)
No	13 (23.2)	7 (12.5)	
Family income			
Poor	8 (14.3)	11 (19.6)	1.992 (0.369)
Moderate	32 (57.1)	35 (62.5)	
Good	16 (28.6)	10 (17.9)	

*Fisher exact test

Table 2 presents the findings with regard to changes on the HBM subscales from baseline to 3-month follow-up after the intervention. All subscales demonstrated significant between-group differences from baseline to follow-up after statistical adjustment by ANCOVA (p < 0.001). However, the greatest change was seen in in the constructs of perceived susceptibility and action (behavior), while domains such as perceived severity and perceived benefits demonstrated only small changes. Within-group comparisons also demonstrated that scores by those in the intervention group improved on all subscales, except for perceived severity and perceived benefits, which worsened from baseline to follow-up. Although some positive change was seen in the control group, the degree of these changes was substantially less than that experienced by intervention group members. When differences between intervention and control groups at baseline and follow up were compared, while there was no significant difference between groups at baseline, the differences at follow-up between intervention and control groups were significant for all dependent variables, favoring the intervention group (p < 0.01).

**Table 2:** Change in HBM constructs in the test and control groups from baseline to 3-month follow-up after the intervention

Measures	Test		Control		Difference (95% CI for difference FL—BL)		Difference (95% CI for difference test—control)		ANCOVA test
	BL (n = 56)	FL (n = 55)	BL (n = 56)	FL (n = 54)					
	Mean (SD)	Mean (SD)	Mean (SD)	Mean (SD)	Test	Control	BL	FL	F (p value)
Perceived susceptibility	6.0 (3.4)	10.2 (1.5)	5.3 (3.1)	5.0 (2.9)	4.2 (3.4–5.1)**	− 0.3 ([− 0.5]–[− 0.1])*	0.7 (− 0.5–1.9)	5.2 (4.3–6.1)**	262.3 (< 0.001)
Perceived severity	24.8 (3.5)	26.1 (2.9)	23.6 (3.8)	24.0 (3.7)	1.3 (0.7–1.9)**	0.4 (0.1–0.7)*	1.2 (− 0.1–2.6)	2.1 (0.9–3.3)*	13.7 (< 0.001)
Perceived benefits	11.0 (1.4)	11.5 (1.3)	10.5 (1.7)	10.4 (1.6)	0.5 (0.2–0.7)*	− 0.1 (− 3.0–[− 0.0])*	0.5 (− 0.1–1.1)	1.1 (0.5–1.7)**	24.4 (< 0.001)
Perceived barriers	15.4 (6.6)	8.5 (5.2)	16.2 (5.8)	16.1 (5.9)	− 6.9 (− 8.2–[− 5.5])**	− 0.1 (− 3.0–[− 0.1])	− 0.8 (− 3.1–1.5)	− 7.6 (− 9.6–[− 5.5])**	137.1 (< 0.001)
Self-efficacy	6.9 (3.4)	10.2 (1.6)	7.7 (3.2)	8.3 (2.8)	3.3 (2.4–4.1)**	0.5 (0.2–0.9)*	− 0.8 (− 2.0–0.4)	1.9 (1.1–2.8)**	53.0 (< 0.001)
Cues to action	7.7 (4.8)	10.2 (2.6)	7.3 (4.1)	7.4 (4.0)	2.5 (1.6–3.6)**	0.1 (-0.0–0.2)	0.4 (− 1.3–2.1)	2.8 (1.6–4.1)**	60.9 (< 0.001)
Action (behavior)	6.4 (2.7)	11.4 (1.2)	6.1 (2.7)	6.7 (2.6)	5.0 (4.2–5.8)**	0.6 (0.4–0.8)**	0.3 (− 0.7–1.3)	4.7 (3.9–5.5)**	226.4 (< 0.001)

*M* mean, *SD* standard deviation, *CI* confidence interval, *BL* baseline, *FL* 3 months follow up, *ANCOVA* analysis of covariance, where the group included as independent variable, baseline measures as covariates and follow-up measures as the dependent variables; **p < 0.001; *p < 0.01

The findings for clinical oral health measures are provided in Table 3. Concerning the ANCOVA results, all measures except for the overall DMFT score demonstrated between-group differences favoring the intervention group after adjusting for baseline differences. The Papillary Bleeding Index (PBI) and decayed teeth demonstrated the largest between-group difference from baseline to follow-up (p < 0.001). However, the between-group difference for the total DMFT score was only minimal (p = 0.105). Computing within-group differences between baseline and follow up on these indices demonstrated significantly improved changes for measures such as PBI, decayed teeth, and filled teeth in the test group, but a worsening was found for missing teeth. While the average number of missing teeth increased in the control group, the mean number of decayed and filled teeth improved. There was less improvement in PBI in the control group compared to the test group from baseline to follow-up (within-group difference = 0.1 vs. 0.7, respectively). There were also significant between-group differences for measures such as PBI, decayed teeth, and some DMFT domain scores (p < 0.05); however, no between-group differences were seen for the overall DMFT score.

**Table 3:** Change in clinical oral health characteristic in the test and control group from baseline to 3-month follow-up

Measures	Test		Control		Difference (95% CI for difference FL—BL)		Difference (95% CI for difference test—control)		ANCOVA test
	BL (n = 56)	FL (n = 55)	BL (n = 56)	FL (n = 54)					
	M (SD)	M (SD)	M (SD)	M (SD)	Test	Control	BL	FL	F (p value)
Papillary Bleeding Index (PBI)	1.4 (1.0)	0.7 (0.6)	1.8 (1.1)	1.7 (1.1)	− 0.7 (− 0.9–[− 0.5])**	− 0.1 (− 0.2–[− 0.0])*	− 0.4 (− 0.8–0.0)*	− 1.0 (− 1.3–[− 0.6])**	44.6 (< 0.001)
Decayed teeth (DT)	3.4 (2.5)	1.9 (1.8)	4.7 (3.4)	4.3 (3.0)	− 1.5 (− 2.0-[− 1.0]**	− 0.4 (− 0.7–[− 0.1])*	− 1.3 (− 2.4–[− 0.1])*	− 2.4 (− 3.3–[− 1.4])**	37.7 (< 0.001)
Missing teeth (MT)	0.8 (1.2)	0.9 (1.2)	0.6 (0.8)	0.9 (0.9)	0.1 (0.0–0.1)*	0.3 (0.2–0.4)**	0.2 (− 0.2–0.6)	− 0.0 (− 0.4–0.4)	10.7 (0.001)
Filled teeth (FT)	1.9 (2.1)	3.2 (2.4)	2.5 (2.3)	2.7 (2.3)	1.3 (0.9–1.6)**	0.2 (0.1–0.3)*	− 6.0 (− 1.4–0.2)	0.5 (− 0.4–1.4)	26.5 (< 0.001)
Total (DMFT)	6.2 (3.5)	6.0 (3.5)	7.8 (4.1)	7.9 (3.7)	− 0.2 (− 0.8–0.5)	0.1 (− 0.2–0.4)	− 1.6 (− 3.0-[− 0.2])*	− 1.9 (− 3.2-[− 0.5])*	2.68 (0.105)

*M* mean, *SD* standard deviation, *BL* baseline, *FL* 3 months follow up, *ANCOVA* analysis of covariance, where the group included as independent variable, baseline measures as covariates and follow-up measures as the dependent variables. **p < 0.001; *p < 0.05

## Discussion

The purpose of this study was to evaluate the impact of an education program based on a psycho-behavioral model of health education (HBM) on oral health behaviors and clinical outcomes in elementary school children. Results indicated a positive impact of the intervention on both psychological constructs of the model as well as on access to restorative treatment for dental caries and improvement of gingival health. Compared to the control group that received only routine oral health education by dental clinic staff, the HBM-based education program taught by a health educator improved significantly more on oral health beliefs, attitudes and behaviors.

Only a few studies have examined HBM-based education programs for improving oral health behaviors in children. In a study by Yekaninejad et al. (2012), researchers attempted to use parents and school staff to improve oral health in school-age children [30]. In that study, 392 children, their parents, and school teachers were enrolled in a randomized controlled trial (RCT) involving three groups: one group receiving a comprehensive HBM-based intervention, one group receiving standard student education, and an untreated control group. In the HBM intervention group, children, their parents and teachers were encouraged to engage in oral health behaviors, whereas children in the student education group received education on oral health and controls received no education. Results indicated that the frequency of oral health behaviors and gingival health improved significantly more in comprehensive HBM group than in the other two groups. In this study, HBM constructs were used to assess the degree of change from baseline to follow-up. Another difference between this HBM educational program and the one provided here is that the present study administered the educational program in the clinic, whereas the latter study provided the educational program in a school setting. Finally, the latter study used a booklet to communicate information on oral health to parents and teachers, whereas the present study was designed to provide face-to-face sessions. Reviewing the results obtained in latter study compared to the current study demonstrated that the present study produced greater changes especially in behavioral aspects when compared to the latter study’s program.

Evidence favoring an HBM-based approach to oral health education in other populations supports the effectiveness of these programs. For example, Malekmohammadi et al. conducted an RCT using an HBM-based educational program to promote oral health for patients with type 2 diabetes [32]. These investigators randomized 120 patients to either intervention or control groups, using the HBM scale on oral health to monitor changes from pretest to posttest. Similar to the findings of the present study, these investigators noted significant changes in all domains of HBM as well as improved oral health behaviors compared to those in the control group. Likewise, Keyong and colleagues used an HBM-based education program for elderly people in Thailand to improve oral health [39]. These investigators followed 162 older adults for 6 months and found that the intervention group (compared to controls) reported better oral health beliefs as well as experiencing lower plaque scores and gingival inflammation. In a third study conducted among adults in the Fars province of Iran, Jeihooni et al. examined the effects of an HBM-based educational program on improving oral health behaviors in pregnant women [31]. In this RCT, 110 women were randomly assigned to intervention or control groups, assessing both groups four months after the intervention. Results indicated significant improvements in oral health beliefs and clinical indicators. Similar to the present study, these investigators used a face-to-face intervention to educate participants. As in the present study, all of these studies found that HBM-based educational interventions improved oral and dental health, justifying the use of this model for oral health education in a wide range of settings and populations.

Other oral health education programs based on psycho-behavioral models to modify the health behaviors of school-age children have reported positive effects as well. For example, Karimy et al. in their evaluation of the Theory of Planned Behavior recruited 356 children ages 11–13 into an RCT where members of the intervention group participated in four one-hour educational sessions conducted over 1 week. Results favored the intervention group, confirming that the use of such education theories/models may increase the effectiveness of oral health education compared to traditional or routine education programs [40]. Likewise, Aleksejuniene et al. examined how an educational program based on social cognitive theory may enhance oral self-care in a sample of adolescents [41]. Using a RCT design, they randomized 197 secondary school children into intervention and control groups, with the intervention group receiving three face-to-face oral health education sessions and assessing outcomes at baseline, 6 months, and 12 months after the intervention. These investigators found that while the 6-month assessment revealed a lower degree of dental plaques in the intervention group compared to controls, this difference disappeared by the 12-month follow-up, suggesting that such benefits may diminish over time, necessitating booster educational sessions to maintain effectiveness. Unfortunately, the current study did not follow up participants for more than three months, leaving open the possibility that the benefits demonstrated here may likewise diminish over time. However, since the current study employed reminders using short text messages for parents that encouraged parents to emphasize to their children the importance of maintaining oral health behaviors, continuing this procedure beyond three months may help to maintain these oral health behaviors over time.

Although use of the DMFT score as an index of oral health in children is recommended by many experts in the field [42], the total score on this index may be less sensitive for determining the benefits of health education programs in short-term studies with limited follow-ups such as the present one. In assessing the effectiveness of the HBM-based educational program here, the components of the DMFT score were innovatively categorized into decayed, missing, and filled teeth separately and changes were assessed in these three components at follow-up. Using this method, it was determined how much decayed teeth were converted to filled or missing teeth and the rate of new decayed teeth. This approach was used previously by our research group when examining the effect of another education program on improvement of oral health in pregnant women using motivational interviewing [43]. In that program, similar to the current results, significant changes were especially evident in terms of reducing decayed teeth. Nevertheless, the total DMFT score continues to be used to evaluate the impact of oral health education in many studies.

One advantage of the current study compared to similar investigations was that both children and a parent were included in educational sessions. This contrasts with most other studies that have focused only on one group (either parents or children). As demonstrated by Yekaninejad et al. [30], this approach may increase the effectiveness of such educational programs especially when implemented in elementary school children, since parents may serve in a supportive role to encourage children to practice oral health behaviors. Also found in the present study was that of the six HBM domains, perceived susceptibility changed the most whereas perceived severity changed the least. The most likely explanation for this finding is that many children and their parents may be aware of the severity of oral health problems, but may feel that they are not at high risk for such conditions. Indeed, participants in the present study, despite their knowledge of such negative oral health outcomes at baseline, did not view themselves as at particular risk for such conditions. However, by the end of the intervention, participants increasingly felt themselves at risk of such oral health problems, which increased their perceived susceptibility scores.

### Study limitations

Although the current study was successful in improving oral health status in school-age children in Iran, several limitations should be noted before implementing such educational programs in a more widespread manner. First, the sample was composed of a middle-income group of Iranians, and it is known that socio-economic status (SES) may have considerable impact on oral health status, thereby limiting the generalization of these results to populations of lower/ higher SES. Future studies examining oral health of children in other countries such as Japan, which mandates oral hygiene during school hours, maybe useful for comparison. Second, including a single dental health center may have affected the generalizability of these findings to all Iranian children. However, given that the present study controlled for external threats to validity, such as selection bias, experimenter effect, and testing effect, it is likely that similar results will be found in other settings as well. Future studies, though, should consider controlling for candy consumption, smoking (if conducted in older children), and other behaviors that may affect oral health and confound the effects of educational interventions. Third, the educational intervention guided by HBM principles provided here was only compared to a control group where participants received routine oral health education program, whereas having more than one intervention group involving other theory-based models of oral health education might identify those that are more effective than the current program. Fourth, due to the nature of the present intervention (education), complete blinding of research personnel and examiners was not possible, thereby leaving open the possibility of bias in assessments. Fifth, an insignificant reduction in the total DMFT score was found in the test group compared to controls, although this may be expected given that each of its components may affect one another. Sixth, participants were not followed for a period longer than three months due to insufficient resources. Therefore, the extent to which beneficial outcomes may persist over time will need to be examined in studies with longer follow-up times. Finally, there are many other valid objective measures other than DMFT score and PBI health, such as plaque, caries, and other oral hygiene indices that could have been used in the present study. However, in order to simplify assessments, they were limited to DMFT and PBI indices as well as the HBM scale. Future studies of educational interventions should consider broadening outcome measures to more comprehensively assess changes in oral health.

## Conclusions

The present study found that an HBM-based program designed to educate children and their parents on oral health prevention may result in improved oral health outcomes such as access to restorative dental treatment, gingival health, and related beliefs and behaviors compared to more traditional clinic-based education. Because many children and their parents may not feel susceptible to oral health problems, using an HBM-based education program may increase awareness of susceptibility to such problems, resulting in better oral health care. Combining educational programs taught in both dental health clinics and at school may help to improve the oral health status in school-age children. Further study of the HBM-based program examined here in different cultural and socioeconomic environments will be useful in determining how these factors may affect the results from such programs. Also, assessment of dental outcomes over longer periods of time is needed to determine how long the benefits of such interventions last.

## Data Availability

Data of this study are not available for public due to confidentiality considerations but the instruments used here are available. For any request of data one may contact the corresponding author.

## Abbreviations

HBM: Health belief model
DMFT: Decayed missing and filled teeth
CI: Confidence interval
WHO: World Health Organization
PBI: Papillary bleeding index
ANCOVA: Analysis of covariance
RCT: Randomized controlled trial
SES: Socio-economic status
